# A Practical Update on Pediatric Eosinophilic Esophagitis

**DOI:** 10.3390/children10101620

**Published:** 2023-09-28

**Authors:** Martina Votto, Maria De Filippo, Silvia Caimmi, Cristiana Indolfi, Alessandro Raffaele, Maria Angela Tosca, Gian Luigi Marseglia, Amelia Licari

**Affiliations:** 1 Pediatric Unit, Department of Clinical, Surgical, Diagnostic and Pediatric Sciences, University of Pavia, 27100 Pavia, Italy; martina.votto@unipv.it (M.V.); maria.defilippo01@universitadipavia.it (M.D.F.); gl.marseglia@smatteo.pv.it (G.L.M.); 2 Pediatric Clinic, Fondazione IRCCS Policlinico San Matteo, 27100 Pavia, Italy; s.caimmi@smatteo.pv.it; 3 Department of Woman, Child and General and Specialized Surgery, University of Campania “Luigi Vanvitelli”, 80138 Naples, Italy; cristiana.indolfi@policliniconapoli.it; 4 Pediatric Surgery Unit, Fondazione IRCCS Policlinico San Matteo, 27100 Pavia, Italy; a.raffaele@smatteo.pv.it; 5 Allergy Center, IRCCS Giannina Gaslini, 16147 Genoa, Italy

**Keywords:** adolescents, allergy, children, eosinophilic esophagitis, food elimination diet, proton pump inhibitor, quality of life, topical steroids

## Abstract

Eosinophilic esophagitis (EoE) is an emerging atopic disease of unknown etiology limited to the esophagus. The pathogenesis is still understood and is likely characterized by type 2 inflammation. Food allergens are the primary triggers of EoE that stimulate inflammatory cells through an impaired esophageal barrier. In children and adolescents, clinical presentation varies with age and mainly includes food refusal, recurrent vomiting, failure to thrive, abdominal/epigastric pain, dysphagia, and food impaction. Upper-gastrointestinal endoscopy is the gold standard for diagnosing and monitoring EoE. EoE therapy aims to achieve clinical, endoscopic, and histological (“deep”) remission; prevent esophageal fibrosis; and improve quality of life. In pediatrics, the cornerstones of therapy are proton pump inhibitors, topical steroids (swallowed fluticasone and viscous budesonide), and food elimination diets. In recent years, much progress has been made in understanding EoE pathogenesis, characterizing the clinical and molecular heterogeneity, and identifying new therapeutic approaches. Notably, clinical, molecular, endoscopic, and histological features reflect and influence the evolution of inflammation over time and the response to currently available treatments. Therefore, different EoE phenotypes and endotypes have recently been recognized. Dupilumab recently was approved by FDA and EMA as the first biological therapy for adolescents (≥12 years) and adults with active EoE, but other biologics are still under consideration. Due to its chronic course, EoE management requires long-term therapy, a multidisciplinary approach, and regular follow-ups.

## 1. Introduction

Eosinophilic esophagitis (EoE) is a chronic/remittent, antigen-mediated disease involving the esophagus [1]. The first case of EoE was described in 1978 by Landres et al. and was considered an esophageal motility disorder [2]. Subsequently, esophageal eosinophilia was considered a feature of gastroesophageal reflux disease (GERD) [3]. EoE was only recognized as a distinct clinical entity by Attwood and Straumann in the early 1990s [4,5]. Since then, several efforts and progress have been made to understand the pathophysiology and natural history of this clinically heterogeneous disease, which significantly impacts patients’ quality of life and health care systems. 

## 2. Epidemiology

EoE has evolved from a rare to a frequent upper gastrointestinal tract disease commonly encountered in pediatric clinical practice [6]. The global prevalence of EoE is 0.5–1 cases/1000 persons [6]. In children, the pooled incidence of EoE is 6.6 cases/100,000 persons each year, whereas the overall prevalence is 34 cases/100,000 children [6]. In the Netherlands, the incidence rates increased from 0.01/100,000 (95% CI: 0.0–0.04) in 1995 to 3.16/100,000 (95% CI: 2.90–3.44) in 2019 [7]. The prevalence of pediatric EoE varies from 2.3/100,000 in Denmark to 90.7/100,000 in Ohio [6]. 

In recent years, several studies have reported a relevant increase in EoE epidemiology, especially in children living in developed countries [8,9,10,11,12,13,14,15]. This phenomenon occurred parallelly with the dramatic increase in the prevalence of allergic disorders observed over the last few decades [16,17]. Economic development combined with high welfare status, the wide distribution of food resources, and improvements in hygienic conditions may potentially contribute to EoE pathogenesis, which is multifactorial. Although some genetic polymorphisms are known to increase the risk of EoE, environmental factors, including a diet rich in modified and enriched foods, are probably the most crucial players in disease development [6,18]. 

## 3. Pathogenesis

EoE is a multifactorial disease in which genes and environment are pathogenetic factors [18]. Their intricate interaction alters the esophageal epithelial barrier, allowing abnormal exposure to allergens (primarily foods) and other luminal components [19]. The impaired barrier leads to the local release of alarmins, including the thymic stromal lymphopoietin (TSLP) and interleukin (IL)-33, which drive the differentiation of T helper 2 (Th2) effector cells and the consequent production of Th2 cytokines (IL-4, IL-5, IL-9, and IL-13) and eosinophil recruitment [20,21]. IgEs, crucial in several atopic diseases, do not have a primary role in EoE pathogenesis [18]. The inflammatory *milieu* in children with active EoE is also characterized by increased angiogenesis, which was demonstrated through evidence of high levels of angiogenic factors, including vascular endothelial growth factor (VEGF), vascular adhesion molecule-1 (VCAM-1), angiogenin, and IL-8 [21].

The role of genetics in EoE pathogenesis was suggested owing to clinical evidence, which has showed that EoE prevalence varies among sex (male: female ratio = 3:1) and ethnicity (EoE is more common in White than Black or Hispanic children) [22,23]. Moreover, having a first-degree family member with EoE is a known risk factor (OR 16.3; 95% CI, 9.4–28.3), which is markedly higher in monozygotic than in dizygotic twins (41% vs. 22%) [24,25]. Despite this evidence, twin studies report a low disease concordance, highlighting the crucial role of environmental factors. The effect of genetics seems to occur in conjunction with environmental factors, including early-life exposures [18]. Early life is a critical period in which the developing immune system and gut microbiota mature and become susceptible to environmental exposures [18]. A few studies have focused on the role of early-life exposures [26,27,28,29,30]. Formula feeding, neonatal intensive care admission, prematurity, maternal fever, early antibiotic or acid suppressant use, and cesarean delivery were all considered putative early risk factors of EoE [18]. 

Genome-wide association (GWA) studies allowed the identification of different genetic loci involved in the expression of Th2 inflammatory cytokines and the regulation and functioning of esophageal epithelial barrier proteins [31,32,33,34]. Desmosomes, tight and adherence junctions, filaggrin, and desmoglein-1 ensure esophageal barrier integrity. Genetic variations in these genes have been reported in EoE patients [35]. The most potent association concerns the expression of dysregulated calpain 14 (CAPN14), an enzyme exclusively expressed in the esophagus and involved in barrier regulation via the IL-13 pathway [31,32,33]. Other polymorphisms were detected in EoE patients and are mainly related to the Th2 immune response, eosinophil chemotaxis, and cell adhesion [31,36,37,38]. GWA studies have also identified other genetic *loci* likely contributing to EoE development, including TSLP, EMSY, LRRC32, STAT6, and ANKRD27 [18,31]. These genetic loci are mainly involved in T helper 2 inflammation and epithelial barrier function and integrity. Interestingly, EoE may complicate the course of different monogenic, inherited diseases. Connective tissue disorders, including Marfan syndrome and Ehlers-Danlos syndromes, share a common pathogenic mechanism through the dysregulation of the TGF-*β* signaling. Children with autosomal dominant hyper-IgE syndrome (HIES) and Netherton syndrome have a high risk of EoE development [39,40]. 

Despite the progress achieved so far, a complete understanding of the molecular pattern will help to further classify patients into endotypes defined by a specific pathophysiologic disease mechanism and to personalize treatments. In this context, using a machine learning approach, Shoda et al. analyzed patients’ histological, endoscopic, and molecular features with EoE, identifying three endotypes. The EoEe1 endotype was recognized in 35% of enrolled patients and was mainly signed by minimal eosinophilic inflammation and responsiveness to topical steroids. The EoEe2 endotype affected 29% of patients, showing prevalent Th2 inflammation, pediatric onset, and low steroid response. The third endotype (EoEe3) was reported in 36% of patients, characterized by adult-onset and strictures [41]. Identifying these endotypes has important clinical implications because they reflect the response to currently available treatments. Indeed, patients with the EoEe1 endotype may be treated with FED and topical steroids, while children with the EoEe2 endotype may benefit from anti-Th2 immune agents (i.e., dupilumab) [42]. The available therapeutic tools for patients with the EoEe3 endotype and strictures are limited; hence, esophageal dilations are the only therapy for esophageal strictures [42]. 

## 4. Diagnosis

EoE is characterized by symptoms of esophageal dysfunction and ≥15 eosinophils per high power field (eos/HPF) in endoscopically obtained biopsies [43]. In patients with esophageal eosinophilia, other causes of esophageal eosinophilia should always be ruled out, particularly GERD, coeliac disease, Crohn’s disease, achalasia, HIES, and drug hypersensitivity. Pediatricians should diagnose EoE based on a combination of symptoms and histological and endoscopic findings, as no single feature is sufficient to establish a definitive diagnosis. Therefore, the essential diagnostic instruments are (1) a detailed medical history, (2) a correct evaluation of endoscopic features, and (3) an accurate histological examination. Upper-gastrointestinal (GI) endoscopy is currently the gold standard for diagnosing and monitoring EoE [43]. Therefore, there is a critical need for noninvasive tools and biomarkers to replace such invasive—but essential—instruments. Despite several efforts to identify potential noninvasive biomarkers, none were included in the guidelines [44,45]. 

Another critical point is that EoE is often delayed or misdiagnosed, especially in the first two decades of life [46]. A longer diagnostic time in children significantly impairs growth and is associated with esophageal tissue remodeling and subepithelial fibrosis, which appear with esophageal rings and strictures [47].

### 4.1. Clinical Features and Heterogeneity of EoE

It has been widely reported that EoE symptoms vary with age [1]. In infants and toddlers, the symptoms of esophageal dysfunction generally appear as feeding difficulties, food refusal, recurrent vomiting, or regurgitation. Older children often report abdominal or epigastric pain and refractory gastroesophageal reflux. Adolescents and adults report dysphagia (first for solid foods, then for liquids) and food impaction episodes [1]. Atypical symptoms have also been reported in EoE patients, such as a recurrent cough in children and heartburn and/or chest pain (including exercise-induced chest pain) in adolescents and adults. Children and adolescents can also develop compensative feeding habits, such as eating slowly, chewing carefully, drinking a lot during meals, cutting food into small pieces, lubricating foods with liquids, and avoiding some foods (meat, bread, and pills) [47]. Adolescents and older children can be worried about eating in public places and thus may develop anxiety disorders. Failure to thrive is a potential complication observed in EoE children due to selective feeding, food refusal, recurrent vomiting, or the occurrence of eating disorders. EoE should be generally suspected in children with gastrointestinal symptoms (reflux, abdominal pain, vomiting) not responsive to conventional therapies, especially if these are related to changes in eating behavior or disorders. Suspicious symptoms or conditions that may help to suspect EoE are summarized in Table 1. 

**Figure 1:**
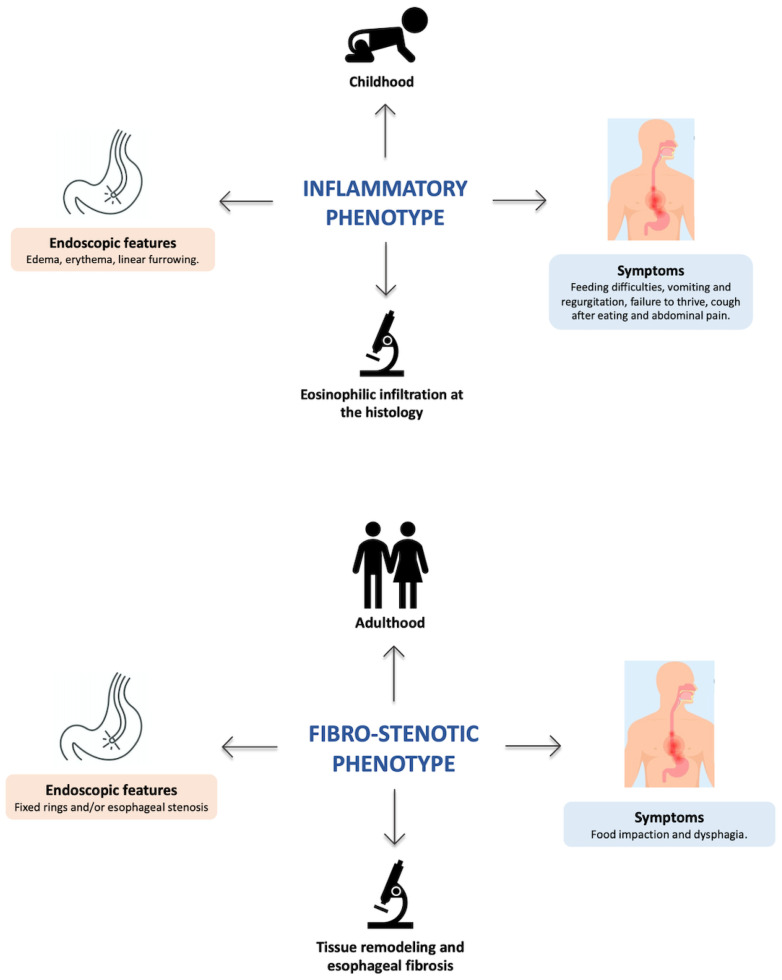
Features of the inflammatory and fibro-stenotic phenotypes.

Notably, clinical, endoscopic, and histological features reflect and influence the evolution of inflammation over time and the response to currently available treatments. Therefore, different EoE phenotypes have been recognized so far [47]. The “inflammatory” phenotype is typically observed in childhood and is characterized by the endoscopic finding of edema, erythema, linear furrowing, and prevalent eosinophilic infiltration found through histology [47,48,49,50] (Figure 1). The “fibro-stenotic” phenotype affects adults, who typically experience dysphagia and food impaction episodes [47,48,49,50]. This phenotype is defined by fixed esophageal rings and/or strictures found through endoscopy and results from tissue-remodeling phenomena [47,48,49,50] (Figure 1). While food elimination diets (FED) and medical therapies may revert esophageal fibrosis in children, this remodeling process may persist despite the resolution of esophageal inflammation in adults [51]. 

Several studies have shown that patients with EoE have concomitant allergic comorbidities, such as allergic rhinitis, asthma, atopic dermatitis, and IgE-mediated food allergy [36]. The prevalence of asthma reaches 60% of prevalence in pediatric series [52]. The prevalence of IgE-mediated food allergy varies from 25% to nearly 70% [53,54]. Eczema was also significantly more frequent in EoE patients than in controls [53,54]. These findings have led many researchers to consider EoE the final step of the atopic march [55]. Conversely, several non-atopic comorbidities are associated with EoE, including inflammatory bowel diseases, connective tissue disorders, autism and attention deficit hyperactivity disorders, esophageal atresia, celiac disease, and the monogenic disorders previously reported [52,56,57]. Eosinophilic gastrointestinal disorders, particularly EoE, have been found in different inborn errors of immunity, including common variable immunodeficiency and HIES [58]. Using a cluster analysis approach, Votto et al. identified and explored the clinical heterogeneity of eosinophilic gastrointestinal disorders (EGIDs), finding two clinical phenotypes of pediatric EoE. Cluster 1 was mainly composed of patients with allergic comorbidities (allergic rhinitis was the prevalent disease), high levels of total serum IgE, and blood peripheral eosinophils. Conversely, Cluster 3 consisted of non-allergic children with a history of neonatal intensive care admission, probably related to the high frequency of congenital malformations observed in this subgroup (such as esophageal atresia) [59].

Recently, Biedermann and colleagues identified and described in adult EoE patients a new clinical syndrome called “food-induced immediate response of the esophagus” or FIRE [60]. FIRE symptoms are highly pronounced, unpleasant, and even painful and are strictly linked to the contact of a specific food trigger with the esophagus, usually appearing a few minutes (<5 min) after ingestion. Symptoms have a limited duration and generally resolve in less than 30 min. The primary identified triggers are fruits, vegetables, and drinks. FIRE might be compared to the pollen food allergy syndrome (PFAS) of the esophagus [60,61]. FIRE has been described in only one pediatric case [62]. The prevalence of FIRE in children may be underestimated because symptoms are challenging to distinguish from EoE, PFAS, and GERD [62].

### 4.2. Endoscopic and Histological Features

Upper-GI endoscopy is the gold standard for EoE diagnosis. In children, esophageal-gastro-duodenoscopy (EGD) is always performed in a hospital setting and with general anesthesia. 

Several endoscopic findings can be recognized in children and adolescents. A graded endoscopic score system has recently been developed to standardize endoscopic findings [63]. The endoscopic reference score (EREFS) is a sum and scoring of the five most prominent endoscopic features of EoE:

- Edema (E), i.e., the loss of vascular markings (present 1, absent 0);
- Rings (R) or esophageal trachealization (none 0, mild 1, moderate 2, severe 3);
- Exudates (E) or white plaques (none 0, mild 1, severe 2);
- Furrows (F) or vertical lines (none 0, mild 1, severe 2);
- Strictures (S) (present 1, absent 0).

Edema, exudates, and furrows are considered inflammatory components, whereas rings and strictures reflect fibrotic components. An EREFS score of less than two corresponds to endoscopic remission. 

Due to the need for repetitive endoscopic evaluations with multiple biopsies and the consequential high costs and psychological burden, minimally invasive methods to diagnose and monitor EoE have recently been proposed. Unsedated transnasal endoscopy was applied in clinical trials; it resulted in a feasible, safe, and cost-effective procedure for children, providing direct visualization of the esophagus and correct acquisition of biopsy samples [64]. Other, less invasive instruments are being validated in pediatric EoE monitoring, such as Cytosponge and endoFLIP to assess mucosal inflammation and esophageal stiffness and motility, respectively.

Unlike other GI tracts, the normal esophagus is entirely devoid of eosinophils. Histologically, ≥15 eosinophils in one HPF are necessary for defining EoE and active disease [43]. Other histological findings have been observed in eosinophilic esophagitis, such as spongiosis (dilation of intercellular spaces), increased mast cell and lymphocyte numbers, basal zone hyperplasia, and papillary elongation [65]. Recently, Collins et al. developed a histological scoring system (EoE-HSS) that analyzes several histologic features: eosinophil density and abscess, basal zone hyperplasia, eosinophil surface layering, dilated intercellular spaces, surface epithelial alteration, dyskeratotic epithelial cells, and lamina propria fibrosis [65]. Although EoE-HSS allows the complete evaluation of all mucosal inflammatory components, its diffusion and application in general and pediatric clinical practice is still limited.

## 5. Therapy

**Figure 2:**
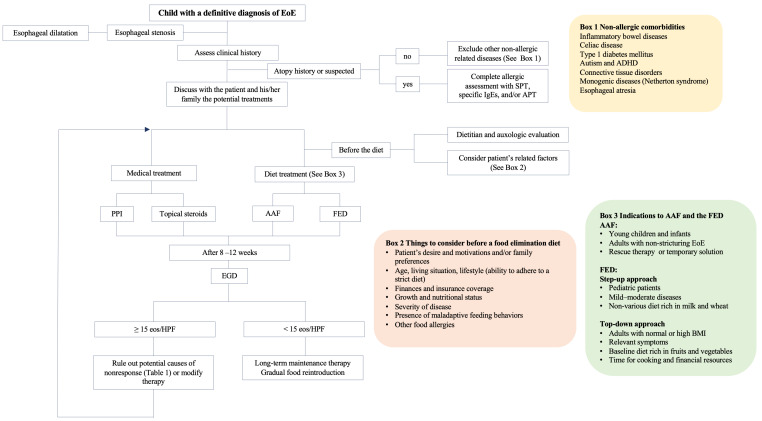
Proposed algorithm for EoE management in children. AAF: aminoacidic based formula; ADHD: attention deficit hyperactivity disorder; ATP: atopy patch test; BMI: body mass index; EGD: esophagogastroduodenoscopy FED: food elimination diet; HPF: high power field; PPI: proton pump inhibitor; SPT: skin prick test.

EoE treatment aims to control symptoms and esophageal inflammation and prevent complications. Current therapeutic options can be distinguished into three categories defined by three Ds: drugs (medical therapy), diet (the elimination of trigger foods), and esophageal dilation [1]. The only currently approved treatment options for EoE are budesonide effervescent tablets, approved for use by adults in most European countries, and dupilumab, approved by the FDA and EMA for patients ≥12 years old [66]. Therefore, treatments routinely used in pediatric clinical practice, like proton pump inhibitors (PPIs) or topical corticosteroids, are not approved for EoE and are prescribed off-label. Choosing the best treatment is not always straightforward and depends on disease-related aspects (severity, the presence of stenosis and comorbidities, nutritional status) but primarily on patient-related factors (the presence of eating and/or mood disorders, financial resources, motivation, lifestyle) (Figure 2). 

### 5.1. Pharmacological Therapy 

#### 5.1.1. Topical Corticosteroids

Topical corticosteroids are effective in inducing EoE remission. Oral steroids can clinically and histologically treat EoE. However, they have significant and well-known side effects if used in the long term. Given the chronicity of EoE, long-term treatment with oral or systemic steroids is not recommended [43]. Meta-analyses of topical corticosteroids demonstrated the superiority of swallowed fluticasone or viscous budesonide compared to a placebo in resolving esophageal eosinophilia, endoscopic findings, and GI symptoms [67,68,69]. Moreover, some evidence has shown that oral budesonide can reverse esophageal fibrosis [70]. Despite regular therapy, the increase in esophageal eosinophil counts has also been described in steroid-refractory or -resistant cases [71,72]. 

There are many unresolved questions about the chronic use of topical steroids. There is no consensus regarding dosage, formulation, frequency, or how to obtain remission using the minimal dose. Long-term side effects are the primary concern, although they have been widely evaluated. Indeed, no studies have been conducted regarding bone health, growth, or adrenal function in children treated with swallowed steroids for longer than one year [73]. The most common side effect of topical steroids is oral and esophageal candidiasis [74]. Thus, further studies are necessary to understand the potential systemic side effects of these therapies in children. 

Topical corticosteroids are typically administered once or twice daily, and dosing depends on age and disease severity (Table 1). Patients should spray fluticasone without a spacer in the back of their mouth and swallow the dose. No eating or drinking is allowed 30 min after administering the medication [73]. 

#### 5.1.2. Proton Pump Inhibitors

The response rates to PPI therapy can vary widely from 30% to 70% [43]. In a meta-analysis of 32 studies on PPI treatment, 50.5% of patients achieved histologic remission [75]. The dosages that are effective in EoE treatment are 1–2 mg/kg in children and 40 mg of omeprazole (or equivalent dosages for other PPIs) once or twice daily in adults (Table 2). The mechanism of action of PPIs in EoE is still unclear. PPIs are well-established inhibitors of H+/K+-ATPase expressed by the gastric parietal cells. Thanks to their pharmacological effect, PPI reduces acidic injury to the esophagus and restores epithelial damage. PPIs also show anti-inflammatory properties, directly inhibiting epithelial STAT6, a key transcription factor for the secretion of pro-inflammatory Th-2 chemokines and cytokines [76,77]. Although the occurrence of PPI use in childhood is higher than that of topical steroids, the possible long-term effects of these medications have not been evaluated in EoE patients. Potential adverse effects (increased risk of fractures, intestinal dysbiosis, or deficiencies in certain micronutrients) should be considered during follow-up. 

#### 5.1.3. Biologic Therapies 

The increasing knowledge of EoE pathogenesis has allowed several therapeutic targets to be identified and tested. The humanized antibodies against IL-5, such as mepolizumab and reslizumab, were tested in three controlled trials in children and adults with active EoE, demonstrating reduced tissue eosinophilia and a favorable safety profile. Unfortunately, clinical improvement was minimal [78,79,80]. A phase III trial using benralizumab, a monoclonal antibody against the IL-5 receptor, remains active [80,81]. 

Two randomized clinical trials (RCTs) with the anti-IL-13 agent and one with the anti-IL-4 receptor antagonist dupilumab showed promising results [82,83]. In a phase II study, a monoclonal antibody against IL-13 improved endoscopic and histological disease activity in the short and long term. Dupilumab is currently the most advanced biologic therapy in EoE treatment. Dupilumab is a humanized monoclonal antibody that binds to the α subunit of the IL-4 receptor and can be found in both IL-4 and IL-13 [84]. In the phase III study, dupilumab significantly relieved symptoms, reduced eosinophil counts, and improved esophageal distensibility [80]. Therefore, the FDA, then the EMA, recently approved dupilumab for treating adolescents (≥12 years) and adults with active EoE. RCTs are active in children younger than 12 years, but preliminary results showed promising results and a good safety profile, with a slight increase in respiratory infections in the treated group compared to the placebo one (Table 3).

### 5.2. Diet Therapies

#### 5.2.1. Elemental Diet

In 1995, Kelly et al. applied the elemental diet (ED) to a small group of children with active EoE, and it was the first effective treatment to be proposed [85]. In the ED, all foods are removed. Patients are fed exclusively with the amino-acid-based formula for at least six to eight weeks [43,86,87]. The ED is the most effective diet therapy [43]. High complete remission rates were reported in children with active disease and an inflammatory phenotype found via endoscopy [88]. Patients with esophageal stenosis had the lowest efficacy rates [89,90,91,92,93]. In children, the ED significantly improves esophageal symptoms and results in a complete histologic remission in about 90% of cases. Therefore, ED is considered a good and valuable therapeutic option in infants and young children, who show the highest treatment compliance (Table 3) [94]. The ED also provides a source of proteins and calories necessary for adequate child growth and puberty spurt, meaning it can be especially proposed for children with EoE triggered by multiple food allergens [94]. The ED is sometimes proposed as a rescue therapy or a “bridge” treatment in adult patients and adolescents with a refractory disease [89,94]. Some authors recently proposed a “modified” ED, adding a few less-allergenic foods (vegetables or fruits) to the diet, thus improving patient acceptance and psychological impact [89,94]. Although the ED is highly effective and induces a rapid remission (of two weeks), unfortunately, its compliance is limited by several disadvantages [95]. The main reasons for low compliance and discontinuation are the unpalatable taste, high costs, and restrictive nature, causing psychosocial isolation for the child [89,94,96]. For these reasons, the ED is not a first-line approach in older children and adolescents, except in severe pediatric cases [94]. 

#### 5.2.2. Empirical Food Elimination Diet (FED)

The FED is the most commonly prescribed diet therapy for EoE. The FED was first proposed to avoid the six primary food triggers of EoE in Western countries. The six-FED (or 6-FED) includes eliminating cow’s milk, wheat/gluten, egg, soy/legumes, peanut/tree nuts, and shellfish/fish [97]. In pediatric studies, the efficacy of 6-FED (assessed according to histologic remission) is about 74% [98]. In childhood, milk is the main food trigger, recognized in up to 85% of cases and followed by wheat/gluten (up to 60% of cases), egg, and soy/legumes. As reported for IgE-mediated food allergy, there is geographic variation in EoE food triggers [88]. Legumes and soy are indeed an uncommon trigger in Spain [88]. Equally, nuts and fish/seafood rarely trigger esophageal inflammation. 

Although 6-FED is less restrictive than the ED, avoiding all six food groups can be challenging. Adherence to 6-FED is often limited by several drawbacks due to the relevant dietary restrictions and the need for frequent EGDs to identify the trigger food(s) and assess disease remission [94]. Therefore, 6-FED is not the ideal treatment option in childhood (Table 4). More recently, less restrictive food elimination diets (removing the most common trigger foods) have been proposed and evaluated. It was demonstrated that most patients who clinically and histologically recover with 6-FED are then allergic to only one or two foods [50]. Kagalwalla et al. reported that the elimination of four (4-FED) foods (cow’s milk, wheat, egg, and soy/legumes) induced histologic remission in 54% of treated children [99,100]. Moreover, children avoiding milk and wheat (2-FED) achieved complete remission in 40% of cases. Finally, adopting a diet free from cow’s milk (1-FED) was effective in 44–51% of pediatric patients [89]. In a recent systematic review with meta-analysis, the overall efficacy of 1-FED was about 70% [98]. In this context, Molina-Infante et al. proposed a step-up approach based on the elimination of one (1-FED) or two (2-FED) more-allergenic foods (generally milk and wheat) [101]. If complete remission is not achieved, the diet is further restricted to four and six foods [101]. This approach should be preferred in children because it leads to faster and easier identification of the culprit food(s), thus reducing the number of EGDs and diet restrictions. 

#### 5.2.3. Allergy-Test-Directed Elimination Diet

IgEs do not have a pathogenetic role in EoE. Thus far, the evidence has demonstrated that food-specific serum IgEs, atopy patch tests, and skin prick tests do not reliably predict food triggers [43,102]. A meta-analysis reported that an allergy-test-directed elimination diet induces histologic remission in about 45% of patients, with higher efficacy rates in children than adults [98]. According to this evidence, current American and European guidelines do not recommend this diet approach [43,102]. 

## 6. EoE Follow-Up

EoE is a chronic disease that requires lifelong therapy and monitoring [43,102]. The absence of guidelines and consensus recommendations limits the long-term management of EoE. In children, follow-up should include the regular assessment of symptoms, growth, nutritional status, endoscopic alterations, and histological abnormalities. Treatment response should be assessed around 8–12 weeks after initiating a novel treatment or after each relevant therapeutical change [103]. Under remission and solid adherence to treatment, the EGD should be performed once per year. 

Patients treated with the FED or ED should be widely informed of the need for several EGDs to assess disease remission when each food or group of foods is reintroduced. Food reintroduction is a long process that requires several months to identify the culprit food(s). Patients treated with 6-FED generally undergo at least six endoscopies [104]. In patients exclusively fed with the amino-acid-based formula, food reintroduction is an even longer process that requires numerous EGDs. 

A recent study demonstrated that, in children, topical steroids can be safely and effectively reduced to the lowest effective dose after a successful induction therapy [105]. 

### 6.1. Nutritional Issues and Assessment

Several factors may negatively influence nutritional status and caloric intake. EoE children generally show GI symptoms, like vomiting or food refusal, that may limit adequate dietary intake [106]. Children and adolescents who experience food impaction episodes present a high risk of developing eating or mood disorders, which may further compromise adequate nutrient and caloric intake [96,107]. The coexistence of multiple food allergies may further complicate the nutritional status and growth of EoE children. Long-term and restrictive food elimination diets may compromise adequate micronutrient intake, although they do not seem to worsen child growth or body mass index (BMI) [106,108]. Nutritionists play an essential role in EoE children’s care. The nutritionist should determine the degree of exposure to EoE trigger foods and the potential nutritional effects of their elimination, meticulously evaluating the patient’s diet and family eating habits [109,110]. Before beginning an empirical food elimination diet and during the follow-up, pediatricians and nutritionists should periodically assess children’s nutritional status and growth and rule out potential nutritional deficiencies. 

Another critical point of EoE follow-up concerns patient and family education. Pediatricians should carefully inform and educate affected patients and families regarding what they can safely eat or cannot eat, avoid cross-contaminations, and provide the appropriate resources for additional information [94]. Parents and children should also be advised on the risk of potential food cross-contamination. Based on the specific local legislation, patients and parents should also be educated and sensitized to interpret foodstuff labels correctly [94]. The precautionary allergen warning (“may contain”) is not obligatory in some countries [94]. The potential risk of trace exposure or allergen contamination is still debated, and it has not been evaluated in EoE patients [94]. 

### 6.2. Multidisciplinary Assessment

**Figure 3:**
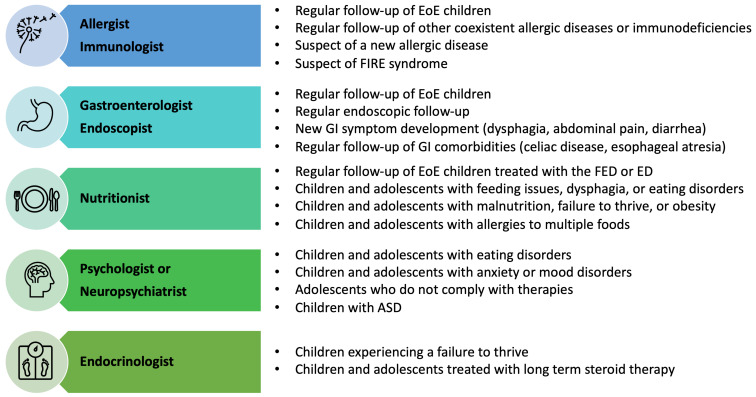
Indications to refer EoE patients to the specialists. ADS, autism spectrum disorders; ED, elemental diet; EoE, eosinophilic esophagitis; FIRE, food-immediate response to the esophagus; FED, food elimination diet.

The multidisciplinary team, including the pediatric allergist, gastroenterologist, endoscopist, nutritionist, and psychologist, is crucial in pediatric EoE management [106] (Figure 3). At highly specialized centers, these pediatric specialists should be enrolled at the first visit and during the follow-up evaluations, guaranteeing the transition from the pediatric to the adult setting. Pediatric allergists should identify and treat coexisting atopic diseases (allergic rhinitis, eczema, asthma, IgE-mediated food allergy, and drug allergy). Allergy assessment is also addressed to stratify the potential risk of IgE-mediated reactions when foods previously eliminated (especially cow’s milk) are reintroduced into the child’s diet [111]. 

Endoscopic and histologic abnormalities are not always associated with gastrointestinal symptoms, and children may be asymptomatic or mildly symptomatic despite active disease. Therefore, endoscopic follow-up is mandatory to evaluate disease remission and patient compliance. Regular multidisciplinary follow-up is also essential to rule out potential side effects of long-term treatments [111]. 

Children with suspected genetic diseases should also be assessed by a medical geneticist. In the case of a suspected hyper-IgE syndrome or Netherton syndrome, the involvement of a pediatric immunologist in the multidisciplinary team is mandatory.

The chronic nature of EoE, comorbidities, long-term therapies, and periodic EGDs are the main stressful factors for children and adolescents with EoE [96]. It has been demonstrated that EoE negatively impacts the QoL of patients [96]. Older children and adolescents may also develop eating disorders or anxiety mostly related to the fear of eating in public places or new food impaction episodes [96]. Therefore, psychological support should be provided when behavioral disorders, eating disorders, or low compliance with therapies are suspected [96]. The psychological impact of EoE is still poorly understood, and more research is needed to assess the burden of neuropsychiatric disorders and their clinical manifestations (sleep disorders, low school performance, dysfunctional family relationships, anxiety, anorexia, or bulimia) and the psychological impact of families.

## 7. Conclusions

EoE is an emerging chronic/remittent allergic disease with a meaningful impact on quality of life and health care systems. Despite several progresses, pediatric EoE management is still limited by gaps and unmet needs. Currently, no treatments have been approved for pediatric EoE management, and studies comparing the superiority of elimination diets with pharmacological therapies or assessing the long-term side effects are still unavailable. EoE follow-up is often limited by numerous drawbacks due to the need for several endoscopies for monitoring the disease, restrictive diets, or long-term pharmacological treatments. Therefore, there is a crucial need to:

1. Personalize treatments according to the molecular profile and clinical features of patients.
2. Assess the long-term effects of currently available therapies.
3. Identify noninvasive biomarkers and new molecular therapeutic targets.
4. Implement the use of less invasive tools to assess disease activity.
5. Improve the diagnostic process to identify the disease and prevent potential complications early.
6. Define international guidelines for long-term pediatric EoE management, focusing on the central role of a multidisciplinary approach.

## Figures and Tables

**Table 1:** Suspicious symptoms of EoE in children.

Infants and Young Children	Older Children	Adolescents
Regurgitation that does not recover with formula thickening, splitting of feedings, or acid suppressants.	GERD-like symptoms that do not recover with acid suppressants.	GERD-like symptoms that do not recover with acid suppressants.
Young children who prefer creamy or smoothed foods, soups, or liquids and avoid solid meals.	Children with selective feeding (avoiding more solid foods like meat and crusty bread).	Dysphagia for solids, then for liquids
Toddlers with speech delay	Children who drink a lot during meals to help food *bolus* progression.	Food impaction episodes
Young children with failure to thrive not related to more common diseases (food allergy, celiac disease, recurrent infections, or other chronic conditions)	Children who eat slowly compared to their siblings or friends.	Eating disorders
Non-surgical causes of recurrent vomiting	Epigastric/abdominal pain that is not responsive to conventional therapies for functional gastrointestinal disorders.	Selective feeding, avoidance of solid food or pills
Non-neurological dysphagia	Episodes of food impaction	Anxiety about eating in public places
Recurrent cough/wheezing	Non-neurological dysphagia for solid food	Adolescents eat slowly compared to their siblings or friends.
Gagging or coughing with feeding	FIRE symptoms	FIRE symptoms
	Recurrent cough	Heartburn or chest pain episodes

FIRE, food-induced immediate response of the esophagus; GERD, gastroesophageal reflux disease.

**Table 2:** Pharmacological therapy of EoE in children and adolescents.

	Proton Pump Inhibitors (PPIs)	Slurry Budesonide	Swallowed Fluticasone	Dupilumab
Dose	Children: 1 mg/kg/day. Adolescents: 20–40 mg/day.	<10 years: 1 mg/day. >10 years: 2 mg/day.	<10 years: 440 mcg twice daily. >10 years: 880 mcg twice daily.	>12 years (weight > 40 kg): 300 mg/weekly.
Specific instructions	Twice daily. 30 min before meals.	Mixed with sucralose (5 g of sucralose), honey, or 2.5 mL aminoacidic formula per mg of budesonide to make a total volume of 8–12 mL.	Do not use the spacer. Do not inhale.	Pre-filled syringe or pre-filled pen for home administration.
General considerations	Initial treatment of 8–12 weeks. Effective in 54% of children.	Second dose administered at bedtime. Avoid eating/drinking 30 min after use. Safe and well tolerated.		Before beginning: ₋ exclude parasitic (helminth) infection; ₋ assess the vaccination status (patients should not receive a live vaccine right before or during treatment); ₋ exclude pregnant status.
Long-term maintenance therapy	PPIs are generally safe. The response is sustained in inflammatory phenotype (70%).	Esophageal candidiasis 4–5%. Consider periodic monitoring for adrenal insufficiency, bone metabolism, and growth. The strategy is to decrease the dose to the lowest adequate level. Limited data on optimal dosage and side effects of long-term use		No long-term data. The most common side effects include injection site reactions, upper respiratory tract infections, cold sores in the mouth or on lips, and joint pain (arthralgia).

PPI, proton pump inhibitor.

**Table 3:** Active trials on biological therapies in children and adolescents with EoE.

NCT Number	Intervention	Population	Status	Phase
NCT04394351	Dupilumab vs. placebo	1–11 years old	Active—not recruiting	Phase 3
NCT05247866	Dupilumab in food reintroduction	6–25 years old	Recruiting	Phase 4
NCT04991935	Cendakimab vs. placebo	12–75 years old	Recruiting	Phase 3
NCT04753697	Cendakimab	12–75 years old	Recruiting	Phase 3
NCT05583227	Tezepelumab vs. placebo	12–80 years old	Recruiting	Phase 3

**Table 4:** Diet treatments of EoE in children and adolescents.

Diet Therapy	Rationale	Indications	Advantages	Disadvantages
Elemental diet (ED)	Exclusive administration of aminoacidic-based formula. Modified ED: one or two less-allergenic foods (vegetables or fruits) are permitted.	Toddlers or young children with severe active EoE. Severe cases. Rescue therapy in severe/refractory cases or temporary solutions.	Rapid remission in 2 weeks. Higher compliance in infants and toddlers. Pediatric amino-acid-based formulas are almost nutritionally complete.	Poor palatability. Low compliance among children and adolescents. Feeding skill regression may be observed in children with NG or G-tube. Amino-acid-based formulas are expensive and not covered by insurance. Less effective in patients with stricturing EoE.
Empiric food elimination diet (FED)	Step-up approach (From 1–2-FED to 6-FED)	Pediatric patients. Moderate symptoms. A diet rich in milk and wheat.	Early identification of trigger food. Short diagnostic process. Avoid unnecessary diet restrictions.	Less effective.
	Top-down approach (From 6-FED to 1–2-FED)	Adults and adolescents with normal or high BMI. Severe symptoms. Baseline diet rich in fruits and vegetables. There is much time to prepare alternative meals and high financial resources.	More effective.	Up to 7 endoscopies, one after every single food reintroduction. Several diet restrictions. Low compliance. Risk of nutritional deficiencies.

BMI, body mass index; ED, elemental diet; FED, food elimination diet; NG, nasogastric.

## Data Availability

Not applicable.

## References

[1] Licari A., Votto M., D’Auria E., Castagnoli R., Caimmi S.M.E., Marseglia G.L. (2020). Eosinophilic Gastrointestinal Diseases in Children: A Practical Review. Curr. Pediatr. Rev..

[2] Landres R.T., Kuster G.G., Strum W.B. (1978). Eosinophilic esophagitis in a patient with vigorous achalasia. Gastroenterology.

[3] Winter H.S., Madara J.L., Stafford R.J., Grand R.J., Quinlan J.E., Goldman H. (1982). Intraepithelial eosinophils: A new diagnostic criterion for reflux esophagitis. Gastroenterology.

[4] Attwood S.E., Smyrk T.C., Demeester T.R., Jones J.B. (1993). Esophageal eosinophilia with dysphagia. A distinct clinicopathologic syndrome. Dig. Dis. Sci..

[5] Straumann A., Spichtin H.P., Bernoulli R., Loosli J., Vögtlin J. (1994). Idiopathic eosinophilic esophagitis: A frequently overlooked disease with typical clinical aspects and discrete endoscopic findings. Schweiz. Med. Wochenschr..

[6] Dellon E.S., Hirano I. (2018). Epidemiology and Natural History of Eosinophilic Esophagitis. Gastroenterology.

[7] de Rooij W.E., Barendsen M.E., Warners M.J., van Rhijn B.D., Verheij J., Bruggink A.H., Bredenoord A.J. (2021). Emerging incidence trends of eosinophilic esophagitis over 25 years: Results of a nationwide register-based pathology cohort. Neurogastroenterol. Motil..

[8] Votto M., Raffaele A., De Filippo M., Caimmi S., Brunero M., Riccipetitoni G., Marseglia G.L., Licari A. (2022). Eosinophilic gastrointestinal disorders in children and adolescents: A single-center experience. Dig. Liver Dis..

[9] Navarro P., Arias Á., Arias-González L., Laserna-Mendieta E.J., Ruiz-Ponce M., Lucendo A.J. (2019). Systematic review with meta-analysis: The growing incidence and prevalence of eosinophilic oesophagitis in children and adults in population-based studies. Aliment. Pharmacol. Ther..

[10] Allin K.H., Poulsen G., Melgaard D., Frandsen L.T., Jess T., Krarup A.L. (2022). Eosinophilic oesophagitis in Denmark: Population-based incidence and prevalence in a nationwide study from 2008 to 2018. United Eur. Gastroenterol. J..

[11] Arias Á., Lucendo A.J. (2019). Incidence and prevalence of eosinophilic oesophagitis increase continiously in adults and children in Central Spain: A 12-year population-based study. Dig. Liver Dis..

[12] Prasad G.A., Alexander J.A., Schleck C.D., Zinsmeister A.R., Smyrk T.C., Elias R.M., Locke III G.R., Talley N.J. (2009). Epidemiology of eosinophilic esophagitis over three decades in Olmsted County, Minnesota. Clin. Gastroenterol. Hepatol..

[13] Hruz P., Straumann A., Bussmann C., Heer P., Simon H.U., Zwahlen M., Beglinger C., Schoepfer A.M. (2011). Escalating incidence of eosinophilic esophagitis: A 20-year prospective, population-based study in Olten County, Switzerland. J. Allergy Clin. Immunol..

[14] van Rhijn B.D., Verheij J., Smout A.J., Bredenoord A.J. (2013). Rapidly increasing incidence of eosinophilic esophagitis in a large cohort. Neurogastroenterol. Motil..

[15] Kapel R.C., Miller J.K., Torres C., Aksoy S., Lash R., Katzka D.A. (2008). Eosinophilic esophagitis: A prevalent disease in the United States that affects all age groups. Gastroenterology.

[16] Thomsen S.F. (2015). Epidemiology and natural history of atopic diseases. Eur. Clin. Respir. J..

[17] Brooks C., Pearce N., Douwes J. (2013). The hygiene hypothesis in allergy and asthma: An update. Curr. Opin. Allergy Clin. Immunol..

[18] Votto M., Marseglia G.L., De Filippo M., Brambilla I., Caimmi S.M.E., Licari A. (2020). Early Life Risk Factors in Pediatric EoE: Could We Prevent This Modern Disease?. Front. Pediatr..

[19] Celebi Sozener Z., Ozdel Ozturk B., Cerci P., Turk M., Gorgulu Akin B., Akdis M., Altiner S., Ozbey U., Ogulur I., Mitamura Y. (2022). Epithelial barrier hypothesis: Effect of the external exposome on the microbiome and epithelial barriers in allergic disease. Allergy.

[20] Zhernov Y.V., Vysochanskaya S.O., Sukhov V.A., Zaostrovtseva O.K., Gorshenin D.S., Sidorova E.A., Mitrokhin O.V. (2021). Molecular Mechanisms of Eosinophilic Esophagitis. Int. J. Mol. Sci..

[21] Ruffner M.A., Kennedy K., Cianferoni A. (2019). Pathophysiology of eosinophilic esophagitis: Recent advances and their clinical implications. Expert Rev. Clin. Immunol..

[22] Liacouras C.A., Spergel J.M., Ruchelli E., Verma R., Mascarenhas M., Semeao E., Flick J., Kelly J., Brown-Whitehorn T., Mamula P. (2005). Eosinophilic esophagitis: A 10-year experience in 381 children. Clin. Gastroenterol. Hepatol..

[23] Mansoor E., Cooper G.S. (2016). The 2010–2015 Prevalence of Eosinophilic Esophagitis in the USA: A Population-Based Study. Dig. Dis. Sci..

[24] Allen-Brady K., Firszt R., Fang J.C., Wong J., Smith K.R., Peterson K.A. (2017). Population-based familial aggregation of eosinophilic esophagitis suggests a genetic contribution. J. Allergy Clin. Immunol..

[25] Alexander E.S., Martin L.J., Collins M.H., Kottyan L.C., Sucharew H., He H., Mukkada V.A., Succop P.A., Abonia J.P., Foote H. (2014). Twin and family studies reveal strong environmental and weaker genetic cues explaining heritability of eosinophilic esophagitis. J. Allergy Clin. Immunol..

[26] Jensen E.T., Kappelman M.D., Kim H.P., Ringel-Kulka T., Dellon E.S. (2013). Early life exposures as risk factors for pediatric eosinophilic esophagitis. J. Pediatr. Gastroenterol. Nutr..

[27] Radano M.C., Yuan Q., Katz A., Fleming J.T., Kubala S., Shreffler W., Keet C.A. (2014). Cesarean section and antibiotic use found to be associated with eosinophilic esophagitis. J. Allergy Clin. Immunol. Pract..

[28] Jensen E.T., Kuhl J.T., Martin L.J., Rothenberg M.E., Dellon E.S. (2018). Prenatal, intrapartum, and postnatal factors are associated with pediatric eosinophilic esophagitis. J. Allergy Clin. Immunol..

[29] Jensen E.T., Gupta S.K. (2018). Early Life Factors and Eosinophilic Esophagitis: Building the Evidence. J. Pediatr. Gastroenterol. Nutr..

[30] Witmer C.P., Susi A., Min S.B., Nylund C.M. (2018). Early Infant Risk Factors for Pediatric Eosinophilic Esophagitis. J. Pediatr. Gastroenterol. Nutr..

[31] O’Shea K.M., Aceves S.S., Dellon E.S., Gupta S.K., Spergel J.M., Furuta G.T., Rothenberg M.E. (2018). Pathophysiology of Eosinophilic Esophagitis. Gastroenterology.

[32] Kottyan L.C., Rothenberg M.E. (2017). Genetics of eosinophilic esophagitis. Mucosal Immunol..

[33] Chang X., March M., Mentch F., Nguyen K., Glessner J., Qu H., Liu Y., Furuta G., Aceves S., Gonsalves N. (2022). A genome-wide association meta-analysis identifies new eosinophilic esophagitis loci. J. Allergy Clin. Immunol..

[34] Sleiman P.M., Wang M.L., Cianferoni A., Aceves S., Gonsalves N., Nadeau K., Bredenoord A.J., Furuta G.T., Spergel J.M., Hakonarson H. (2014). GWAS identifies four novel eosinophilic esophagitis loci. Nat. Commun..

[35] Chang J.W., Jensen E.T., Dellon E.S. (2022). Nature with Nurture: The Role of Intrinsic Genetic and Extrinsic Environmental Factors on Eosinophilic Esophagitis. Curr. Allergy Asthma Rep..

[36] Rossi C.M., Lenti M.V., Merli S., Licari A., Votto M., Marseglia G.L., Di Sabatino A. (2022). Primary eosinophilic gastrointestinal disorders and allergy: Clinical and therapeutic implications. Clin. Transl. Allergy.

[37] Capucilli P., Hill D.A. (2019). Allergic Comorbidity in Eosinophilic Esophagitis: Mechanistic Relevance and Clinical Implications. Clin. Rev. Allergy Immunol..

[38] Blanchard C., Mingler M.K., McBride M., Putnam P.E., Collins M.H., Chang G., Stringer K., Abonia J.P., Molkentin J.D., Rothenberg M.E. (2008). Periostin facilitates eosinophil tissue infiltration in allergic lung and esophageal responses. Mucosal Immunol..

[39] Arora M., Bagi P., Strongin A., Heimall J., Zhao X., Lawrence M.G., Trivedi A., Henderson C., Hsu A., Quezado M. (2017). Gastrointestinal Manifestations of STAT3-Deficient Hyper-IgE Syndrome. J. Clin. Immunol..

[40] Paluel-Marmont C., Bellon N., Barbet P., Leclerc-Mercier S., Hadj-Rabia S., Dupont C., Bodemer C. (2017). Eosinophilic esophagitis and colonic mucosal eosinophilia in Netherton syndrome. J. Allergy Clin. Immunol..

[41] Shoda T., Wen T., Aceves S.S., Abonia J.P., Atkins D., Bonis P.A., Caldwell J.M., Capocelli K.E., Carpenter C.L., Collins M.H. (2018). Eosinophilic oesophagitis endotype classification by molecular, clinical, and histopathological analyses: A cross-sectional study. Lancet Gastroenterol. Hepatol..

[42] Keely S., Talley N.J. (2018). Endophenotyping eosinophilic oesophagitis: A new era for management?. Lancet Gastroenterol. Hepatol..

[43] Dellon E.S., Liacouras C.A., Molina-Infante J., Furuta G.T., Spergel J.M., Zevit N., Spechler S.J., Attwood S.E., Straumann A., Aceves S.S. (2018). Updated International Consensus Diagnostic Criteria for Eosinophilic Esophagitis: Proceedings of the AGREE Conference. Gastroenterology.

[44] Votto M., De Filippo M., Castagnoli R., Delle Cave F., Giffoni F., Santi V., Vergani M., Caffarelli C., De Amici M., Marseglia G.L. (2021). Noninvasive biomarkers of eosinophilic esophagitis. Acta Biomed..

[45] Grueso-Navarro E., Navarro P., Laserna-Mendieta E.J., Lucendo A.J., Arias-González L. (2023). Blood-Based Biomarkers for Eosinophilic Esophagitis and Concomitant Atopic Diseases: A Look into the Potential of Extracellular Vesicles. Int. J. Mol. Sci..

[46] Votto M., Lenti M.V., De Silvestri A., Bertaina F., Bertozzi M., Caimmi S., Cereda E., De Filippo M., Di Sabatino A., Klersy C. (2023). Evaluation of diagnostic time in pediatric patients with eosinophilic gastrointestinal disorders according to their clinical features. Ital. J. Pediatr..

[47] Muir A.B., Brown-Whitehorn T., Godwin B., Cianferoni A. (2019). Eosinophilic esophagitis: Early diagnosis is the key. Clin. Exp. Gastroenterol..

[48] Ruffner M.A., Cianferoni A. (2020). Phenotypes and endotypes in eosinophilic esophagitis. Ann. Allergy Asthma Immunol..

[49] Hirano I., Sharaf R., Stollman N., Wang K., Falck-Ytter Y., Chan E., Rank M., Stukus D., Greenhawt M. (2020). Spotlight: Treatment of Eosinophilic Esophagitis (EoE). Gastroenterology.

[50] Steinbach E.C., Hernandez M., Dellon E.S. (2018). Eosinophilic Esophagitis and the Eosinophilic Gastrointestinal Diseases: Approach to Diagnosis and Management. J. Allergy Clin. Immunol. Pract..

[51] Shaheen N.J., Mukkada V., Eichinger C.S., Schofield H., Todorova L., Falk G.W. (2018). Natural history of eosinophilic esophagitis: A systematic review of epidemiology and disease course. Dis. Esophagus.

[52] Capucilli P., Cianferoni A., Grundmeier R.W., Spergel J.M. (2018). Comparison of comorbid diagnoses in children with and without eosinophilic esophagitis in a large population. Ann. Allergy Asthma Immunol..

[53] González-Cervera J., Arias Á., Redondo-González O., Cano-Mollinedo M.M., Terreehorst I., Lucendo A.J. (2017). Association between atopic manifestations and eosinophilic esophagitis: A systematic review and meta-analysis. Ann. Allergy Asthma Immunol..

[54] Hill D.A., Dudley J.W., Spergel J.M. (2017). The Prevalence of Eosinophilic Esophagitis in Pediatric Patients with IgE-Mediated Food Allergy. J. Allergy Clin. Immunol. Pract..

[55] Hill D.A., Grundmeier R.W., Ramos M., Spergel J.M. (2018). Eosinophilic Esophagitis Is a Late Manifestation of the Allergic March. J. Allergy Clin. Immunol. Pract..

[56] Talathi S., Knight T., Dimmitt R., Mestre J., Jester T. (2019). Concurrent eosinophilic esophagitis in pediatric patients with inflammatory bowel disease: A case series. Ann. Allergy Asthma Immunol..

[57] Abonia J.P., Wen T., Stucke E.M., Grotjan T., Griffith M.S., Kemme K.A., Collins M.H., Putnam P.E., Franciosi J.P., von Tiehl K.F. (2013). High prevalence of eosinophilic esophagitis in patients with inherited connective tissue disorders. J. Allergy Clin. Immunol..

[58] Votto M., Naso M., Brambilla I., Caimmi S., De Filippo M., Licari A., Marseglia G.L., Castagnoli R. (2023). Eosinophilic Gastrointestinal Diseases in Inborn Errors of Immunity. J. Clin. Med..

[59] Votto M., Fasola S., Cilluffo G., Ferrante G., La Grutta S., Marseglia G.L., Licari A. (2022). Cluster analysis of clinical data reveals three pediatric eosinophilic gastrointestinal disorder phenotypes. Pediatr. Allergy Immunol..

[60] Biedermann L., Holbreich M., Atkins D., Chehade M., Dellon E.S., Furuta G.T., Hirano I., Gonsalves N., Greuter T., Gupta S. (2021). Food-induced immediate response of the esophagus-A newly identified syndrome in patients with eosinophilic esophagitis. Allergy.

[61] Holbreich M., Straumann A. (2021). Features of food-induced immediate response in the esophagus (FIRE) in a series of adult patients with eosinophilic esophagitis. Allergy.

[62] Vott M., Naso M., De Filippo M., Marseglia A., Raffaele A., Marseglia G.L., Licari A. (2022). Food-induced immediate response of the esophagus: A first report in the pediatric age. Allergy.

[63] Hirano I., Moy N., Heckman M.G., Thomas C.S., Gonsalves N., Achem S.R. (2013). Endoscopic assessment of the oesophageal features of eosinophilic oesophagitis: Validation of a novel classification and grading system. Gut.

[64] Venkatesh R.D., Leinwand K., Nguyen N. (2023). Pediatric Unsedated Transnasal Endoscopy. Gastrointest. Endosc. Clin. N. Am..

[65] Collins M.H., Martin L.J., Alexander E.S., Boyd J.T., Sheridan R., He H., Pentiuk S., Putnam P.E., Abonia J.P., Mukkada V.A. (2017). Newly developed and validated eosinophilic esophagitis histology scoring system and evidence that it outperforms peak eosinophil count for disease diagnosis and monitoring. Dis. Esophagus.

[66] Biedermann L., Straumann A., Greuter T., Schreiner P. (2021). Eosinophilic esophagitis-established facts and new horizons. Semin. Immunopathol..

[67] Murali A.R., Gupta A., Attar B.M., Ravi V., Koduru P. (2016). Topical steroids in eosinophilic esophagitis: Systematic review and meta-analysis of placebo-controlled randomized clinical trials. J. Gastroenterol. Hepatol..

[68] Rawla P., Sunkara T., Thandra K.C., Gaduputi V. (2018). Efficacy and Safety of Budesonide in the Treatment of Eosinophilic Esophagitis: Updated Systematic Review and Meta-Analysis of Randomized and Non-Randomized Studies. Drugs R D.

[69] Dellon E.S., Sheikh A., Speck O., Woodward K., Whitlow A.B., Hores J.M., Ivanovic M., Chau A., Woosley J.T., Madanick R.D. (2012). Viscous topical is more effective than nebulized steroid therapy for patients with eosinophilic esophagitis. Gastroenterology.

[70] Aceves S.S., Newbury R.O., Chen D., Mueller J., Dohil R., Hoffman H., Bastian J.F., Broide D.H. (2010). Resolution of remodeling in eosinophilic esophagitis correlates with epithelial response to topical corticosteroids. Allergy.

[71] Eluri S., Runge T.M., Hansen J., Kochar B., Reed C.C., Robey B.S., Woosley J.T., Shaheen N.J., Dellon E.S. (2017). Diminishing Effectiveness of Long-Term Maintenance Topical Steroid Therapy in PPI Non-Responsive Eosinophilic Esophagitis. Clin. Transl. Gastroenterol..

[72] Collins C.A., Palmquist J., Proudfoot J.A., Qian A., Wangberg H., Khosh-Hemmat E., Khosh-Hemmat E., Dohil R., Aceves S.S. (2019). Evaluation of long-term course in children with eosinophilic esophagitis reveals distinct histologic patterns and clinical characteristics. J. Allergy Clin. Immunol..

[73] Cianferoni A. (2020). Eosinophilic esophagitis and other eosinophilic disorders of the gastrointestinal tract. Pediatr. Allergy Immunol..

[74] Jensen E.T., Huang K.Z., Chen H.X., Landes L.E., McConnell K.A., Almond M.A., Safta A.M., Johnston D.T., Durban R., Jobe L. (2019). Longitudinal Growth Outcomes Following First-line Treatment for Pediatric Patients with Eosinophilic Esophagitis. J. Pediatr. Gastroenterol. Nutr..

[75] Lucendo A.J., Arias Á., Molina-Infante J. (2016). Efficacy of Proton Pump Inhibitor Drugs for Inducing Clinical and Histologic Remission in Patients with Symptomatic Esophageal Eosinophilia: A Systematic Review and Meta-Analysis. Clin. Gastroenterol. Hepatol..

[76] Wen T., Dellon E.S., Moawad F.J., Furuta G.T., Aceves S.S., Rothenberg M.E. (2015). Transcriptome analysis of proton pump inhibitor-responsive esophageal eosinophilia reveals proton pump inhibitor-reversible allergic inflammation. J. Allergy Clin. Immunol..

[77] Zhang X., Cheng E., Huo X., Yu C., Zhang Q., Pham T.H., Wang D.H., Spechler S.J., Souza R.F. (2012). Omeprazole blocks STAT6 binding to the eotaxin-3 promoter in eosinophilic esophagitis cells. PLoS ONE.

[78] Straumann A., Conus S., Grzonka P., Kita H., Kephart G., Bussmann C., Beglinger C., Smith D.A., Patel J., Byrne M. (2010). Anti-interleukin-5 antibody treatment (mepolizumab) in active eosinophilic oesophagitis: A randomized, placebo-controlled, double-blind trial. Gut.

[79] Spergel J.M., Rothenberg M.E., Collins M.H., Furuta G.T., Markowitz J.E., Fuchs G., O’Gorman M.A., Abonia J.P., Young J., Henkel T. (2012). Reslizumab in children and adolescents with eosinophilic esophagitis: Results of a double-blind, randomized, placebo-controlled trial. J. Allergy Clin. Immunol..

[80] Biedermann L., Straumann A. (2023). Mechanisms and clinical management of eosinophilic oesophagitis: An overview. Nat. Rev. Gastroenterol. Hepatol..

[81] Hassani M., Koenderman L. (2018). Immunological and hematological effects of IL-5(Rα)-targeted therapy: An overview. Allergy.

[82] Rothenberg M.E., Wen T., Greenberg A., Alpan O., Enav B., Hirano I., Nadeau K., Kaiser S., Peters T., Perez A. (2015). Intravenous anti-IL-13 mAb QAX576 for the treatment of eosinophilic esophagitis. J. Allergy Clin. Immunol..

[83] Hirano I., Collins M.H., Assouline-Dayan Y., Larry E., Gupta S.K., Straumann A., Safroneeva E., Grimm M., Smith H., Tompkins C.A. (2016). A randomized, double-blind, placebo-controlled trial of a novel recombinant, humanized, anti-interleukin-13 monoclonal antibody (RPC4046) in patients with active eosinophilic esophagitis: Results of the HEROES study. United Eur. Gastroenterol. J..

[84] Licari A., Castagnoli R., Marseglia A., Olivero F., Votto M., Ciprandi G., Marseglia G.L. (2020). Dupilumab to Treat Type 2 Inflammatory Diseases in Children and Adolescents. Paediatr. Drugs.

[85] Kelly K.J., Lazenby A.J., Rowe P.C., Yardley J.H., Perman J.A., Sampson H.A. (1995). Eosinophilic esophagitis attributed to gastroesophageal reflux: Improvement with an amino acid-based formula. Gastroenterology.

[86] Lucendo A.J., Molina-Infante J., Arias Á., von Arnim U., Bredenoord A.J., Bussmann C., Amil Dias J., Bove M., González-Cervera J., Larsson H. (2017). Guidelines on eosinophilic esophagitis: Evidence-based statements and recommendations for diagnosis and management in children and adults. United Eur. Gastroenterol. J..

[87] De Bortoli N., Penagini R., Savarino E., Marchi S. (2017). Eosinophilic esophagitis: Update in diagnosis and management. Position paper by the Italian Society of Gastroenterology and Gastrointestinal Endoscopy (SIGE). Dig. Liver Dis..

[88] Molina-Infante J., Lucendo A.J. (2018). Dietary therapy for eosinophilic esophagitis. J. Allergy Clin. Immunol..

[89] Chehade M., Aceves S.S. (2021). Treatment of Eosinophilic Esophagitis: Diet or Medication?. J. Allergy Clin. Immunol. Pract..

[90] Henderson C.J., Abonia J.P., King E.C., Putnam P.E., Collins M.H., Franciosi J.P., Rothenberg M.E. (2012). Comparative dietary therapy effectiveness in remission of pediatric eosinophilic esophagitis. J. Allergy Clin. Immunol..

[91] Markowitz J.E., Spergel J.M., Ruchelli E., Liacouras C.A. (2003). Elemental diet is an effective treatment for eosinophilic esophagitis in children and adolescents. Am. J. Gastroenterol..

[92] Warners M.J., Vlieg-Boerstra B.J., Verheij J., van Rhijn B.D., Van Ampting M.T., Harthoorn L.F., de Jonge W.J., Smout A.J., Bredenoord A.J. (2017). Elemental diet decreases inflammation and improves symptoms in adult eosinophilic oesophagitis patients. Aliment. Pharmacol. Ther..

[93] Peterson K.A., Byrne K.R., Vinson L.A., Ying J., Boynton K.K., Fang J.C., Gleich G.J., Adler D.G., Clayton F. (2013). Elemental diet induces histologic response in adult eosinophilic esophagitis. Am. J. Gastroenterol..

[94] Cianferoni A., Shuker M., Brown-Whitehorn T., Hunter H., Venter C., Spergel J.M. (2019). Food avoidance strategies in eosinophilic oesophagitis. Clin. Exp. Allergy.

[95] Visaggi P., Mariani L., Pardi V., Rosi E.M., Pugno C., Bellini M., Bellini M., Zingone F., Ghisa M., Marabotto E. (2021). Dietary Management of Eosinophilic Esophagitis: Tailoring the Approach. Nutrients.

[96] Votto M., Castagnoli R., De Filippo M., Brambilla I., Cuppari C., Marseglia G.L., Licari A. (2020). Behavioral issues and quality of life in children with eosinophilic esophagitis. Minerva Pediatr..

[97] Kagalwalla A.F., Sentongo T.A., Ritz S., Hess T., Nelson S.P., Emerick K.M., Melin-Aldana H., Li B.U. (2006). Effect of six-food elimination diet on clinical and histologic outcomes in eosinophilic esophagitis. Clin. Gastroenterol. Hepatol..

[98] Arias A., González-Cervera J., Tenias J.M., Lucendo A.J. (2014). Efficacy of dietary interventions for inducing histologic remission in patients with eosinophilic esophagitis: A systematic review and meta-analysis. Gastroenterology.

[99] Kagalwalla A.F., Wechsler J.B., Amsden K., Schwartz S., Makhija M., Olive A., Davis C.M., Manuel-Rubio M., Marcus S., Shaykin R. (2017). Efficacy of a 4-Food Elimination Diet for Children with Eosinophilic Esophagitis. Clin. Gastroenterol. Hepatol..

[100] Molina-Infante J., Arias A., Barrio J., Rodríguez-Sánchez J., Sanchez-Cazalilla M., Lucendo A.J. (2014). Four-food group elimination diet for adult eosinophilic esophagitis: A prospective multicenter study. J. Allergy Clin. Immunol..

[101] Molina-Infante J., Arias Á., Alcedo J., Garcia-Romero R., Casabona-Frances S., Prieto-Garcia A., Modolell I., Gonzalez-Cordero P.L., Perez-Martinez I., Martin-Lorente J.L. (2018). Step-up empiric elimination diet for pediatric and adult eosinophilic esophagitis: The 2-4-6 study. J. Allergy Clin. Immunol..

[102] Hirano I., Chan E.S., Rank M.A., Sharaf R.N., Stollman N.H., Stukus D.R., Wang K., Greenhawt M., Falck-Ytter Y.T., Chachu K.A. (2020). AGA Institute and the Joint Task Force on Allergy-Immunology Practice Parameters Clinical Guidelines for the Management of Eosinophilic Esophagitis. Gastroenterology.

[103] Greuter T., Alexander J.A., Straumann A., Katzka D.A. (2018). Diagnostic and Therapeutic Long-term Management of Eosinophilic Esophagitis- Current Concepts and Perspectives for Steroid Use. Clin. Transl. Gastroenterol..

[104] Lucendo A.J. (2015). Meta-Analysis-Based Guidance for Dietary Management in Eosinophilic Esophagitis. Curr. Gastroenterol. Rep..

[105] Oliva S., Volpe D., Russo G., Veraldi S., Papoff P., Giordano C., Ruggiero C., Trovato C.M., Terrin G., Rossetti D. (2022). Maintenance Therapy with the Lowest Effective Dose of Oral Viscous Budesonide in Children with Eosinophilic Esophagitis. Clin. Gastroenterol. Hepatol..

[106] Votto M., De Filippo M., Olivero F., Raffaele A., Cereda E., De Amici M., Testa G., Marseglia G.L., Licari A. (2020). Malnutrition in Eosinophilic Gastrointestinal Disorders. Nutrients.

[107] Mehta H., Groetch M., Wang J. (2013). Growth and nutritional concerns in children with food allergy. Curr. Opin. Allergy Clin. Immunol..

[108] Colson D., Kalach N., Soulaines P., Vannerom Y., Campeotto F., Talbotec C., Chatenoud L., Hankard R., Dupont C. (2014). The impact of dietary therapy on clinical and biologic parameters of pediatric patients with eosinophilic esophagitis. J. Allergy Clin. Immunol. Pract..

[109] Mehta P., Furuta G.T., Brennan T., Henry M.L., Maune N.C., Sundaram S.S., Menard-Katcher C., Atkins D., Takurukura F., Giffen S. (2018). Nutritional State and Feeding Behaviors of Children with Eosinophilic Esophagitis and Gastroesophageal Reflux Disease. J. Pediatr. Gastroenterol. Nutr..

[110] Groetch M., Venter C., Skypala I., Vlieg-Boerstra B., Grimshaw K., Durban R., Cassin A., Henry M., Kliewer K., Kabbash L. (2017). Dietary Therapy and Nutrition Management of Eosinophilic Esophagitis: A Work Group Report of the American Academy of Allergy, Asthma, and Immunology. J. Allergy Clin. Immunol. Pract..

[111] Votto M., De Filippo M., Lenti M.V., Rossi C.M., Di Sabatino A., Marseglia G.L., Licari A. (2022). Diet Therapy in Eosinophilic Esophagitis. Focus on a Personalized Approach. Front. Pediatr..

